# In Memory of Dr. Taba, the Legendary WHO Regional Director

**DOI:** 10.34172/aim.2024.33

**Published:** 2024-04-01

**Authors:** Yasmine Motarjemi

**Affiliations:** ^1^Food Safety Consultant, Nyon, Switzerland

**Keywords:** Dr. Motarjemi, Dr. Taba, EMRO, Health, Iran, Public health, World Health Organization

## Abstract

The article is a tribute to Dr. Abdul-Hossein Tabatabai-Naini, the former Regional Director of the Eastern Mediterranean Regional Office (EMRO), on the occasion of WHO’s 75th anniversary. It reports on his achievements, personality, and philosophy of medicine.

## Introduction

 On January 28, 2024, the Eastern Mediterranean Regional Office of the World Health Organization (WHO/EMRO)celebrated the 75th anniversary of WHO with the theme “Treasuring the past, painting a healthier and safer future”.

 During the celebrations, which focused on WHO’s past achievements and future challenges, the leadership and dedication of former WHO/EMRO Regional Directors^[1]^ were recognized and their contribution to public health commended.

 Here follows a tribute to Dr. Abdul-Hossein Tabatabai-Naini, known as Dr. Taba (Iran, 1911–1982).

 The celebration of the 75th anniversary of WHO, in the Eastern Mediterranean Regional Office (EMRO), has brought back many memories of the late Dr. Taba, the WHO Regional Director.

 I, his stepdaughter, would like to share some of them for the record.

 At the time I met Dr. Taba through my mother, I was a young student and unfortunately did not have the opportunity or the wisdom to get to know him better in his professional capacity. However, his career path, his achievements, and his contribution to public health are duly documented by Mrs. Leila Mounesan and Dr. Ehsan Mostafavi.^1^ We learn there that Dr. Taba’s contribution to public health extends from the promotion of primary healthcare, the control of malaria, schistosomiasis and diarrheal diseases, to the eradication of smallpox, the development of programs to combat non-communicable diseases and to promote health education services.

 In an article published in the Lancet, Dr. Taba is described as an advanced and open-minded person whose thinking has influenced the 24 countries in the region. It also states that medical education, in the sense of health education, was one of his main interests. The number of medical schools in the EMR region is said to have risen from a handful to nearly a hundred. He launched an extensive fellowship program and took a personal interest in every fellow sponsored by EMRO.^2^ His philosophy of medicine and health comes across in a speech he gave when he was awarded an honorary doctorate in medicine by the University of Birmingham in 1976. He advises the new health professionals to study “*not just man himself, his molecular, biological make-up but man in the widest senses, in the context of his total environment”.*^3^

 However, a person is more than his work. Here, I would like to comment on his personal qualities. Some may have perceived him as a severe and distant person. A WHO representative once said, “*he is aristocratic and autocratic but always right*.”

 As a stepdaughter who had the pleasure of getting to know him from the inside, I kept a different image of him. I remember him as a charismatic person with a soft and warm smile. If I had to summarize my memories of him in a few words, the first word that would come to my mind would be ‘respect.’ He commanded respect in every way. He respected others, and, in return, he was greatly respected and admired.^1,4^

 It is reported that at the time he was elected Regional Director, it was thought that his term of office would be short-lived due to the ongoing anti-Iranian campaign. Yet, he was unanimously elected for several terms from 1957 to 1982; overall, he served WHO for 30 years. The newspaper Kayhan International writes of him: “*The fact that Dr. Taba has managed to bypass the politics of regional states is solely and entirely due to his strong sense of integrity and humanitarianism, as well as his high standards of professionalism*.”^4^ Indeed, although he had the love of his country, Iran, at heart and was a true nationalist, he served the region and its people with loyalty and integrity.

 The newspaper also reports on his dedication and commitment to his work. When the Arab-Israeli Six-Day War broke out, Dr. Taba was in London. Egyptian airports were closed, and traveling to the region was highly risky. Nevertheless, Dr. Taba flew to Libya and then took the long, arduous road to Alexandria to get to his office. It took him three days. He wanted to be behind his desk in case WHO needed him.^4^

 I witnessed eight years of his life with my mother, Mrs Touran Talischi Heravi. He never spoke harshly to anyone, never raised his voice. He was kind and generous to his personnel. He was disciplined to a degree I’ve rarely seen. Punctual, poised, measured, and perhaps meticulous. That is how I saw him and remember him. Besides being a proficient surgeon and public health professional, he mastered several languages.

 He was a loving husband to my mother and a caring father to his daughter, Farah. He was elegant and set an example of a healthy lifestyle. He was careful about his diet and played several sports, including golf and, in his youth, tennis. While committed to assuming his professional responsibilities, he also liked to have fun and fully enjoy life. He embodied and symbolized health, and *world health was his business*.^4^

 I would also like to say a few words about my relationship with him. I am one of the most fortunate people on this planet as, besides having a loving mother and family, my father, Dr. Homayoun Motarjemi (Iran, 1926–1981),^5^ and stepfather fill my heart with warmth and affection. They both symbolized “*Ensâniat*,” which means “humanity” in Persian. They have both been a source of inspiration in my professional life. Their memory has given me the strength to practice my profession with humanity, ethics, and dignity. I feel very fortunate to have known Dr. Taba and had him in my life.

 It is comforting to know that my mother and Dr. Taba found happiness, and their love never faded. My mother continues to cherish his memory, and there is not a day that goes by that she does not remember him dearly.

 You understand that the memory of such a person can never be forgotten. So, let me share two memories of Dr. Taba that always make me smile.

 The first is the time Dr. Taba met my mother. It is a memory that tells us that, despite his commitment to his work, he considered love part of life and essential to health and happiness. It was September 1973 in Ramsar, a city in northern Iran, at a conference. I attended the conference’s final evening for a social event. To return to Tehran, the capital, Dr. Taba arranged for my mother and me to travel in his car. My mother and Dr. Taba sat in the back seat, and I sat beside the driver. By this time, they were already attracted to each other but were too shy or cautious to express it. Throughout the journey, which lasted at least three hours, my mother and Dr. Taba talked about nothing except the trees we passed by. They commented on every single tree. All along the way, I smiled at their disguised flirting.

 The other memory is when we had guests for dinner. In keeping with his healthy lifestyle, Dr. Taba used to get to bed early, around 10 o’clock. So, when guests stayed late, Dr. Taba would get up and roll down the blinds to signal that it was time to leave. Some guests never got it.

 Finally, apart from his contribution to public health and his achievements, one of Dr. Taba’s most precious legacies is the many friends and loyal colleagues he left behind, whose friendship we continue to treasure.

## Endnotes


^[1]^Dr. Aly Shousha (1949–1957); Dr. Abdul-Hossein Taba (1957–1982); Dr. Hussein Gezairy (1982–2012); Dr. Ala Alawan (2012–2017, Dr. Mohammad Fikri, 2017 Dr, Jaouad Mahjour (2017–2018), Dr. Ahmad Al-Mandhari (2018–2023).

## References

[1] Mounesan L, Mostafavi E (2020). In Honor of Dr Abdul Hussein Taba: Iranian Physician and Former Director of the Eastern Mediterranean Region of the World Health Organization. Arch Iran Med.

[2] Anon. Abdul Hossein Taba. The Lancet, July 7. 1982.

[3] Anon. Speech by the Royal Society of Medicine at the occasion of Honorary Fellow-ship award. London, 18 July 1978.

[4] A Healthy World Is His Business, by Bizhan Irandoust. Keyhan International, October 4, 1967.

[5] Motarjemi Y. Infomeduse. Tribute to My Father! January 21, 2021. Available from: https://www.infomeduse.ch/2021/01/21/hommage-a-mon-pere/.

